# Perioperative Nutrition in Pediatric Patients with Congenital Heart Disease and Heart Failure

**DOI:** 10.3390/nu17223609

**Published:** 2025-11-19

**Authors:** Jaime Silva-Gburek, Kelsey May, Bailey Walvoord, Catalina Lozano, Jorge A. Coss-Bu

**Affiliations:** 1 Pediatric Critical Care Medicine, Children’s Mercy Hospital, University of Missouri-Kansas City School of Medicine, Kansas City, MO 64108, USA; 2 Hospital Zambrano Helión, Escuela de Medicina Ignacio Santos, TecSalud, Monterrey 66278, Mexico; 3 Division of Critical Care Medicine, Department of Pediatrics, Baylor College of Medicine, Houston, TX 77030, USA

**Keywords:** congenital heart disease, enteral nutrition, parenteral nutrition, indirect calorimetry, resting energy expenditure, extracorporeal membrane oxygenation, ventricular assist device, cardiac necrotizing enterocolitis

## Abstract

Optimal nutritional therapy is important for infants and children with perioperative congenital heart disease and heart failure. Medical providers face physiological and metabolic challenges when administering enteral and parenteral nutrition to these patients. Complications related to enteral nutrition can increase morbidity and mortality, although outcomes are improved in those cases in which caloric and nutrient administration can be achieved. Consensus regarding feeding strategies and timing of nutritive care is lacking. This narrative review aims to summarize, analyze and discuss the most recent literature on nutritional therapy in perioperative congenital heart disease, heart failure and pediatric patients requiring mechanical circulatory support in the intensive care unit. We also present our own institution’s enteral feeding protocols and rationale for their use supported by evidence-based medicine.

## 1. Introduction

From the time Hippocrates stated, “Let thy food be thy medicine and thy medicine be thy food” and Harvey discovered that the heart pumps blood throughout the human body [1], it has been recognized that nutrition and cardiovascular function play a transcendental role in health. However, despite more than 2000 years of medical practice, many mysteries remain regarding the optimal nutritional therapy for pediatric cardiac patients.

What we do know is that congenital cardiac malformations are one of the most common congenital anomalies [2,3,4]. Also, we know that almost half of these patients will suffer from malnutrition as a result of a high metabolic rate, challenges in the adequate provision of calories and nutrients, gastrointestinal malabsorption, difficulties in accurately measuring basal energy requirement, and an inflammatory state secondary to cardiopulmonary bypass and heart failure [5,6,7,8,9]. Finally, we know that poor nutritional status in congenital heart disease (CHD) cases leads to worse clinical outcomes such as longer duration of mechanical ventilation, extended hospital length of stay, infectious complications, poor neurological development, and higher perioperative mortality [10,11,12,13,14,15,16,17,18].

Fortunately, although the techniques and practices for providing nutritional therapy to cardiac patients remain quite variable [19,20,21,22], it has been demonstrated that implementing feeding protocols in cardiac intensive care units and optimizing nutritional status can significantly improve clinical outcomes [23,24,25,26,27]. In the following narrative review, we will summarize the most recent medical literature on nutritional therapy, the most common gastrointestinal complications, and the most appropriate dietary techniques to promote growth, development, and survival in pediatric patients with CHD, heart failure and those requiring mechanical circulatory support. We also present our own institutions specific enteral feeding protocols and rationale for their use supported by evidence-based medicine.

## 2. Methods

A comprehensive literature search was conducted covering all publications available across PubMed, Scopus, Embase, and the Cochrane Library databases. Medical Subject Headings (MeSH) used in the search included: pediatrics, critically ill, nutrition, heart failure and CHD. The search was limited to articles between 2000 and 2025. Conference abstracts, case reports, editorials, and non-English articles were excluded unless an English abstract was available. Review articles were also examined in detail to identify additional relevant studies. Two reviewers screened all titles and abstracts to identify relevant studies. All selected articles aligned with the objectives of this narrative review and were approved by all authors (see Supplemental Materials).

## 3. Energy Requirements and Nutritional Evaluation

Estimating caloric requirements in these patients is a challenging task, as cardiopulmonary bypass, cardiovascular surgery, inflammation, pulmonary overcirculation, and hemodynamic instability produce variable and sometimes contradictory effects on metabolic rate [6,28,29,30]. The American Society for Parenteral and Enteral Nutrition (ASPEN) and the European Society of Paediatric and Neonatal Intensive Care (ESPNIC) recommend the use of indirect calorimetry (IC) as the gold standard for determining resting energy expenditure (REE) [31,32]. However, the regular implementation of this technology in the cardiac intensive care unit may be limited, as its accuracy is affected by the use of inhaled nitric oxide, high fraction of inspired oxygen, extracorporeal membrane oxygenation (ECMO), and small tidal volumes in neonates [33]. In such cases, or when access to IC equipment is unavailable, predictive equations should be used. A systematic review found no significant difference between the fidelity of the Harris–Benedict, Schofield, or WHO equations in the CHD population [34].

The nutritional assessment of critically ill cardiac patients also presents a unique challenge. Body weight and other anthropometric measurements may be falsely elevated secondary to fluid overload as a result of cardiac failure. In addition, nutritional biomarkers such as albumin and prealbumin may be significantly affected by renal failure, liver failure, cardiopulmonary bypass, and intravenous albumin use, which are common in this population [35,36,37]. Soon, novel technologies such as the use of musculoskeletal ultrasound and bioelectrical impedance may emerge as more accurate alternatives to traditional methods of nutritional assessment in the cardiac intensive care unit [38,39,40]. Until then, accurate, precise, and consistent evaluation tools are lacking.

In addition to anthropometric measurements and innovative biomarkers, the development of nutrition screening tools may also help detect patients with malnutrition admitted to critical care units. Such tools would aid in focusing limited nutritional resources towards at risk patients that need them the most. Although some tools have showed promise [41,42] there unfortunately has yet to be developed a validated nutrition screening tool that reliably identifies these patients. As of yet, none have been directly recommended by ASPEN or ESPNIC to be utilized consistently in the pediatric cardiac critical care population [31,32].

## 4. Preoperative Nutritional Support

Most neonates with CHD have intrauterine growth within normal ranges [43]. However, due to the high metabolic rate of these patients, difficulties in achieving appropriate caloric intake, presence of cyanosis, large left-to-right shunts, heart failure, and coexistence of non-cardiac malformations, neonates become drastically malnourished soon after birth [44,45]. The incidence of malnutrition in infants with unrepaired CHD is approximately 40% [46], and regrettably, this figure has not shown signs of improvement in the last 3 decades [35].

Fortunately, it has been observed that patients undergoing surgical repair who have an adequate level of nutrition experience a shorter duration of mechanical ventilation, a shorter hospital length of stay, less organ dysfunction, and lower surgical mortality [47,48]. So significant is the effect of nutritional therapy on surgical outcomes that in patients with hypoplastic left heart syndrome (HLHS) even only administering pre-surgical trophic feeds (10–20 mL/kg/day) is sufficient to decrease the duration of mechanical ventilation, decrease fluid overload, and promote tolerance to early post-surgical enteral nutrition [49]. In neonates who do not tolerate the enteral route before surgical repair, there is evidence that simply performing oral care with colostrum can enrich and strengthen their immune system [50,51,52,53].

**Figure 1:**
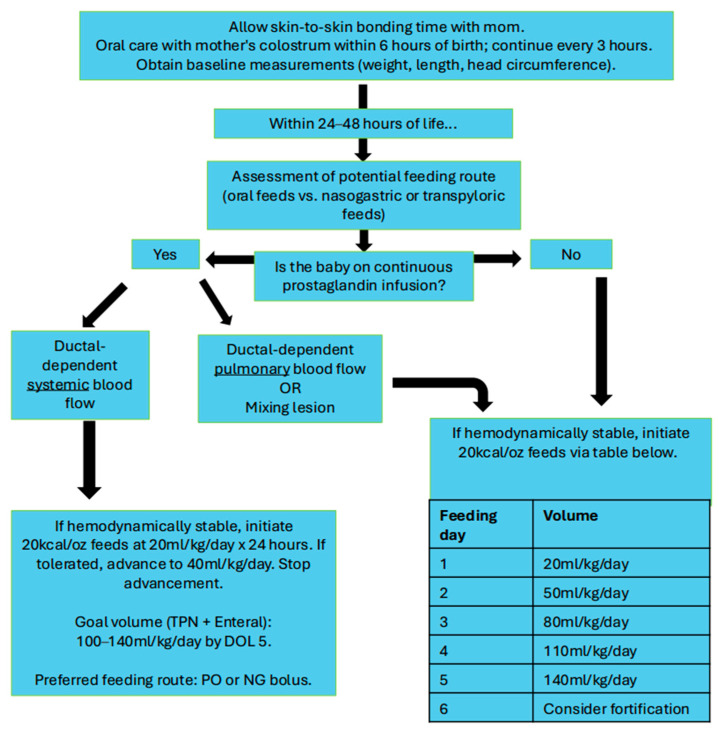
Preoperative feeding protocol for newborns with critical congenital heart disease. Adapted from the protocols and clinical guidelines of the cardiac intensive care unit of Children’s Mercy Hospital, Kansas City, MO, USA. TPN, total parenteral nutrition; DOL, day of life; PO, per os; NG, nasogastric.

The decision to provide preoperative enteral nutrition depends on factors such as the direction of ductal flow, the use of prostaglandins, and the use of inotropic and vasopressor infusions [22]. Currently, there is no consensus on the optimal method for providing preoperative enteral nutrition to neonates with CHD, and significant variability exists in the clinical practices of each institution. A study by the Pediatric Cardiac Critical Care Consortium (PC4) demonstrated that only about 50% of neonates received preoperative enteral nutrition, and the range of infants being fed before surgery varied from 29% to 79% among the clinical centers studied [19]. Although there is much inconsistency in clinical practice, several recent studies have demonstrated that preoperative enteral nutrition is safe [54,55,56,57]. Furthermore, the Neonatal Cardiac Care Collaborative (NeoC3) recently stated that “there is insufficient evidence to conclude that preoperative feeding adversely affects perioperative clinical outcomes” [58]. Below, we present our institutions preoperative neonatal feeding protocol for newborns with CHD (Figure 1).

### 4.1. Ductal-Dependent Heart Disease

In theory, a neonate with ductal-dependent heart disease (receiving prostaglandin infusion or in the presence of ductal stenting) would suffer a high risk of mesenteric hypoperfusion due to diastolic steal, making them more prone to suffering cardiac NEC [59]. It was previously believed that ductal-dependent heart disease was a contraindication for initiating preoperative enteral nutrition. In the Single Ventricle Reconstruction Trial (2005–2008), only 12% of these patients received enteral nutrition before surgery [60]. However, a study conducted 10 years later showed that currently, up to 42% of these newborns receive nutritional therapy via the enteral route [19]. This significant change is likely due to the growing body of medical evidence, including randomized trials, that demonstrate this clinical practice is safe and beneficial [49,54,55,61]. Several retrospective studies, as well as a meta-analysis, have not found a relationship between preoperative enteral nutrition in ductal-dependent heart disease and the development of cardiac NEC [57,62,63,64]. Currently, ESPNIC and NeoC3 affirm that preoperative enteral nutrition is safe in these patients as long as they are hemodynamically stabile and have adequate clinical monitoring [31,58].

### 4.2. Presence of Arterial Umbilical Catheter

The presence of an arterial umbilical catheter is another scenario that could theoretically increase the risk of cardiac NEC. The possibility of decreased blood flow through the mesenteric vessels due to the relative obstruction of the abdominal aorta and the potential for mesenteric arterial thrombosis are reasons why intensivists and dieticians in the past were extremely cautious about initiating enteral nutrition in these cardiac patients. However, Doppler studies have found no differences in flow patterns or vascular resistances in mesenteric vessels in neonates with arterial umbilical catheters [65,66]. In addition, retrospective studies of more than 600 patients with CHD have not demonstrated an association between the presence of an arterial umbilical catheter and risk of cardiac NEC [19,67,68]. As in the case of ductal-dependent heart disease, ESPNIC and NeoC3 concur that enteral nutrition is safe in the presence of an arterial umbilical catheter, provided the patient is hemodynamically stable and under adequate clinical monitoring [31,58].

## 5. Postoperative Nutritional Support

As with preoperative nutrition, there is significant variability in the implementation of postoperative nutritional therapy in pediatric cardiac patients. The progression of this therapy is highly dependent on hemodynamic stability, the use of vasoactive medications, fluid restrictions, and the respiratory support the patient is receiving [40,43]. A multicenter study found that the mean time to initiate feeds in this population ranges from 1 to 4 postoperative days [19]. Other studies have shown that some patients wait more than a month to receive enteral nutrition, and even for those who initiate feeds within the first three postoperative days, less than half receive more than 100 kcal/kg/day [69,70]. What is known is that postoperative nutrition in patients with heart disease is safe, more beneficial when started early, and that feeding protocols serve to accelerate the progression of feeding, reduce the need for parenteral nutrition and reduce the risk of cardiac NEC [20,23,24,25,26,31,61,65,71,72,73].

Prolonged fasting is associated with intestinal mucosal atrophy and can lead to bacterial translocation, sepsis, and multiple organ dysfunction [74,75]. It is recommended to initiate enteral nutritional therapy as early as possible, ideally within the first 24–48 h postoperatively. A randomized controlled study found that infants with cyanotic heart disease who received trophic feeds (10–20 mL/kg/day) 4–6 h after surgery did not experience a higher incidence of gastrointestinal complications and exhibited a shorter duration of mechanical ventilation and a shorter hospital stay [76]. Other studies in critically ill children have shown that early feeding decreases the incidence of nosocomial infections, reduces muscle wasting, and promotes wound healing [77,78,79]. Clinical guidelines from the Society of Critical Care Medicine (SCCM), ESPNIC and the ASPEN recommend initiating enteral nutrition orally or through a nasogastric tube as these methods are safe, more closely resemble the normal physiology of a pediatric patient and promote the secretion of postprandial hormones that promote intestinal development [31,32,80]. However, there is some evidence that nasoduodenal tube feeding decreases the risk of aspiration [31,32], decreases interruptions to enteral nutritional therapy [81], and can even be continued throughout the process of extubation [82,83].

Although there are no prospective studies specifically in patients with CHD, several retrospective studies in children and adults have reported that enteral nutrition is safe, even during the administration of vasopressor drugs [31,81,84]. The most recent SCCM and ASPEN clinical guidelines recommend the use of enteral nutritional therapy in critically ill pediatric patients, provided the patient demonstrates signs of adequate cardiac output and the infusion dose of vasopressor medications is not increasing [32]. This suggestion is also applicable to patients with cardiac conditions.

Vocal cord dysfunction is a well-recognized complication of cardiac surgery. The recurrent laryngeal nerve is prone to surgical injury due to its proximity to the aortic arch [40,85]. It is recommended that any patient with CHD who undergoes surgery involving the aorta have a fiberoptic evaluation of vocal cord function by an otolaryngologist before starting oral feeds because of the high risk of aspiration. More recently, some investigators have found that laryngeal ultrasound also has a high sensitivity and specificity in the diagnosis of this surgical complication [86,87].

**Figure 2:**
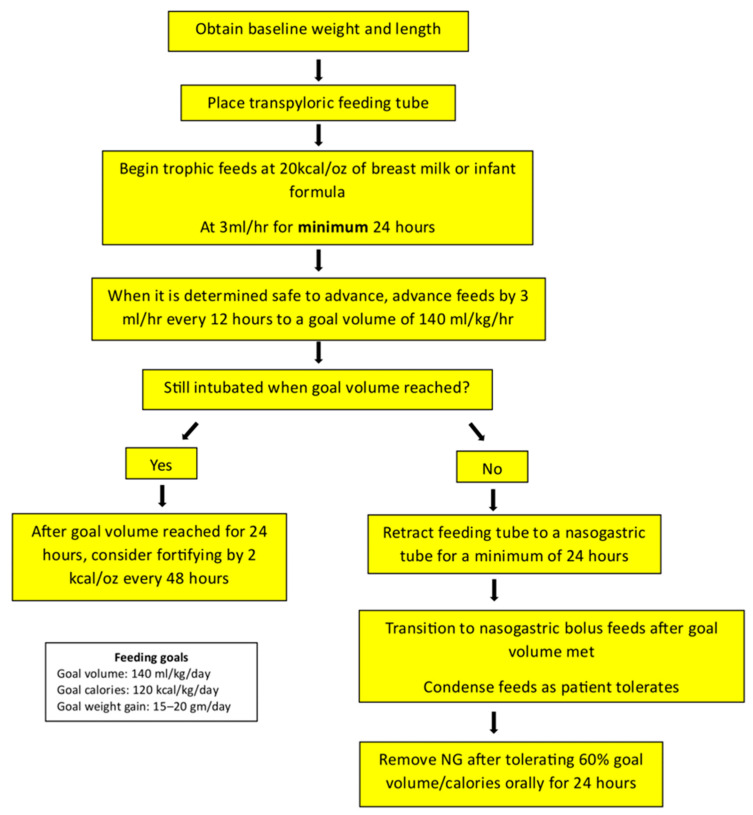
Postoperative feeding protocol for newborns with congenital heart disease. Adapted from the protocols and clinical guidelines of the cardiac intensive care unit of Children’s Mercy Hospital, Kansas City, MO, USA.

It is recommended that each institution establish a feeding protocol for its postoperative cardiovascular surgery patients, as there is abundant evidence that using clinical algorithms in the cardiac intensive care unit reduces variability in clinical practice and improves outcomes [23,25]. Additionally, the multidisciplinary participation of dieticians, nurses, and advanced practice providers in decision-making regarding postoperative nutritional therapy is highly beneficial. More research is needed to determine which protocol is the most effective and safe. Below, we present our institution’s postoperative feeding protocol for newborns with CHD (Figure 2).

It is not precisely known how post-surgical metabolic stress or cardiopulmonary bypass affects patients’ basal energy requirement. Previously, it was believed that these factors produced a hypermetabolic state. However, more recent studies have shown that the energy requirement can vary dramatically between patients [30,88] and does not normalize until the seventh postoperative day [89]. It is due to this metabolic uncertainty that indirect calorimetry is recommended to estimate nutritional requirements during this clinical stage [58].

## 6. Parenteral Nutrition

There is significant controversy regarding when to use parenteral nutrition in critically ill pediatric patients. Unfortunately, the European Society of Parenteral and Enteral Nutrition (ESPEN), ASPEN, and ESPNIC do not have specific clinical guidelines for patients with CHD [31,32,90]. However, parenteral nutrition is a tool commonly used in the cardiac intensive care unit, as these patients have high energy requirements, prolonged fasting periods, hemodynamic instability, a high risk of mesenteric ischemia, and often suffer from oral intolerance [40].

A subanalysis of the neonatal cohort of the PEPaNIC study (within which 35% of the patients had been admitted after cardiac surgery) found that early administration of parenteral nutrition (first 24 h of admission) was associated with higher incidence of nosocomial infections and longer duration of mechanical ventilation and hospital stay [91]. However, only 25% of the cardiac patients in this study received late parenteral nutrition, which may significantly limit the generalizability and clinical applicability of these results.

Despite the findings of the PEPaNIC study, infants with heart disease are often prescribed early parenteral nutrition [40,58,79]. In the cardiac intensive care unit of Children’s Mercy Hospital, every newborn with CHD with ductal-dependent systemic blood flow or those at high risk for cardiac NEC are started on parenteral nutrition within 48 h of life (Figure 1). The goal is to achieve 80–100 mL/kg/day of nutrition via the venous route within 5 days of life, while awaiting cardiovascular surgery. In the postoperative period, the administration of parenteral nutrition is considered at 48 h postoperatively in patients with hemodynamic instability, particularly those receiving high doses of continuous infusions of vasoactive or inotropic medications, or those who cannot tolerate the enteral route. A central venous catheter should always be available when using this nutritional therapy in patients with CHD, allowing for the administration of higher caloric concentrations and a reduction in the total volume of fluids administered [40,92].

## 7. Special Considerations

### 7.1. Heart Failure and Ventricular Assist Device

Like children with CHD, pediatric patients with heart failure also present a unique clinical challenge when implementing nutritional therapy. Systolic and diastolic dysfunction increase metabolic demand and fluid restriction hinders the delivery of required calories. Additionally, elevated central venous pressure leads to gastrointestinal edema, which reduces the absorption of essential nutrients. The Pediatric Cardiomyopathy Registry found that 23.7% of children with dilated cardiomyopathy suffer from malnutrition [93]. Optimizing nutrition and weight gain in these patients has a pronounced effect on their clinical outcomes, including mortality. Recent studies have shown that having a body mass index (BMI) below the 1st percentile increases the risk of pre- and post-heart transplant mortality and decreases the likelihood of graft survival [94,95,96,97].

Patients with heart failure and malnutrition require consultation with a specialized dietician. It is essential to identify any deficiencies in micronutrients, such as iron, vitamin D, and carnitine [98]. The use of hypercaloric formulas, the addition of fats in food, and the use of nasogastric or nasoduodenal tubes are techniques that can promote growth. In some severe cases, supplementation with parenteral nutrition is also required [98].

Thanks to advances in biomedical technology, clinicians now have access to a vital tool in the fight against malnutrition in patients with CHD and/or heart failure: the ventricular assist device (VAD). These devices, which can be either intracorporeal or extracorporeal, utilize continuous or pulsatile flow to generate cardiac output. Their presence in the world of pediatrics has expanded exponentially over the last decade, and now VADs can be used in patients weighing as little as two kilograms [99,100].

**Figure 3:**
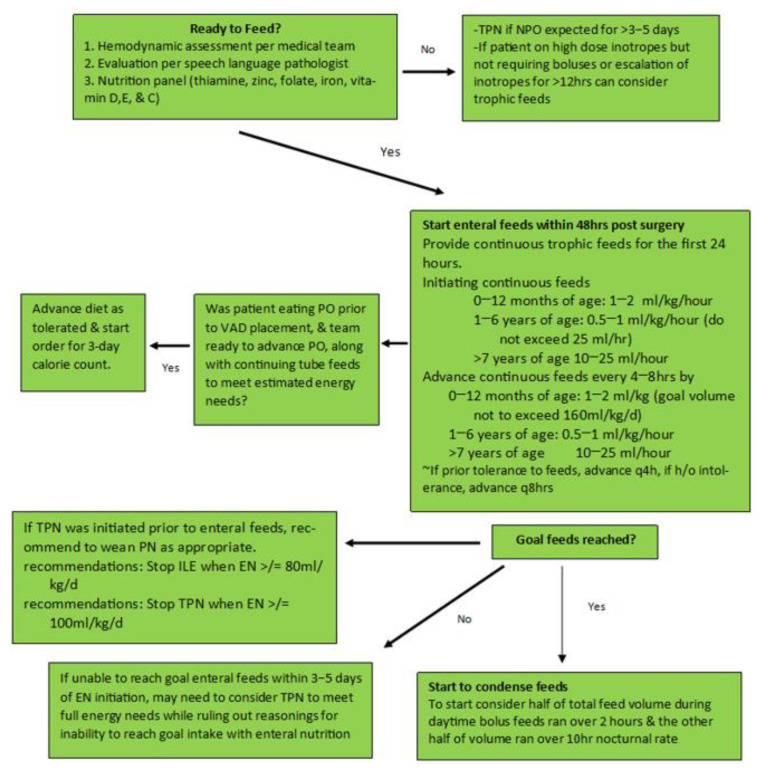
Postoperative ventricular assist device implantation feeding protocol. Adapted from the protocols and clinical guidelines of the cardiac intensive care unit of Children’s Mercy Hospital, Kansas City, MO, USA. TPN, total parenteral nutrition; VAD, ventricular assist device; PN, parenteral nutrition; ILE, intralipid emulsion; EN, enteral nutrition.

After VAD implantation, the increase in caloric intake ranges from 77% to 90%, and the proportion of patients with malnutrition decreases significantly [101]. Some clinical trials have found that VAD insertion enables nutritional rehabilitation in pediatric patients with heart failure, improves oral feeding tolerance, and facilitates an increase in BMI [97,102,103,104]. Restarting enteral nutrition immediately after VAD placement in the postoperative period is of utmost importance. Currently, there is no validated protocol describing this process. However, most experts recommend initiating trophic feeds through a nasogastric or nasoduodenal tube in the first 24–48 h post-surgery (Figure 3). Medical providers should consider starting parenteral nutrition during this period if the patient was admitted with a diagnosis of malnutrition and should strive to reach a goal of 80% of the nutritional requirement between the 3rd and 5th postoperative day [105].

### 7.2. Extracorporeal Membrane Oxygenation

The most recent studies have shown that up to 3% of pediatric patients admitted to a cardiac intensive care unit will require extracorporeal membrane oxygenation (ECMO) support [106] and 33% of this population will suffer from malnutrition [107]. These patients tend to be supported with vasoactive medications, require severe fluid restriction, and suffer from multiple organ dysfunction [40].

One of the biggest challenges is determining the REE of these children on mechanical circulatory support. Classically, it has been recommended to use IC to determine this requirement in critically ill patients. However, since there are two gas exchange systems present (the lungs and the extracorporeal oxygenation membrane), IC tends to significantly underestimate the REE [108]. This is why several investigators have described creative techniques to simultaneously connect the metabolic cart to the mechanical ventilator and the oxygenation membrane [109]. These techniques have also been successfully applied to pediatric patients [110]. These and similar clinical trials have found that the REE in ECMO patients is highly variable and ranges from hypermetabolic to hypometabolic states [30,109,110,111,112]. Therefore, there are no specific universal recommendations for this group of patients; however, we do have clinical guidelines that help describe the most recommended nutritional therapies.

ASPEN and NeoC3 have determined that enteral nutrition in ECMO-supported patients is safe [58,113]. ASPEN also recommends initiating trophic feeds within the first 24–48 h, provided clinical stability is established. A retrospective study found that any type of enteral nutrition administered within the first 5 days of ECMO was associated with improved survival [113,114]. Attempts should be made to provide 80% of REE for the first week, and consideration should be given to initiating parenteral nutrition if 50% of this goal is not achieved via the enteral route by the first 3–5 ECMO days [113]. ESPNIC also recommends providing at least 1.5 g/kg/day of protein [31]. Despite the existence of these clinical guidelines, nutritional therapy in ECMO patients remains highly variable, and in very few patients is an appropriate caloric intake achieved [115,116,117]. It is recommended that each institute should have its own nutrition protocol for ECMO patients to help prioritize optimal nutritional therapy and promote positive clinical outcomes.

### 7.3. Chylothorax

Chylothorax is the accumulation of lymph in the pleural space and can be a post-operative complication in cardiac patients, particularly when there is damage to the thoracic duct or an increase in central venous pressure. It occurs in 1.3–5% of patients and is more frequent after operations involving the aortic arch [118,119,120,121]. Chylothorax is also more common in patients with Turner and Noonan Syndrome [121]. Due to the high protein content of lymph, prolonged and high-volume chylothorax can lead to coagulopathies, immune system dysfunction, poor wound healing, electrolyte disturbances, impaired cardiac output, and malnutrition [43]. This, in turn, leads to prolonged use of mechanical ventilation, a longer hospital length of stay, increased medical costs, and higher postoperative mortality [120,122].

There is considerable variability in the management of this surgical complication, and, to date, no randomized controlled trial has been performed to determine the best clinical practice [40]. For the time being, it is recommended to follow the clinical guidelines of the PC4 Chylothorax Work Group, which recommend starting with a fat-modified diet for 24–72 h [123]. This diet may consist of medium-chain triglyceride formulas or reduced-fat human milk (fortified with protein). If the chest tube volume is less than 20 mL/kg/day, it is recommended to continue this nutritional therapy for 2–4 weeks. If the volume exceeds 20 mL/kg/day, it is recommended to initiate a period of fasting with total parenteral nutrition support for 1 week [123]. If these interventions do not resolve the chylothorax, more invasive therapies, such as percutaneous embolization of the thoracic duct, pleurodesis, continuous infusion of octreotide, or surgical ligation of the thoracic duct, should be considered [43,121].

## 8. Gastrointestinal Complications

### 8.1. Cardiac Necrotizing Enterocolitis

Cardiac NEC is characterized by intestinal inflammation, disruption of mucosal integrity, and bacterial translocation [79]. Unlike necrotizing enterocolitis of prematurity, which is caused by immaturity of the digestive tract, cardiac NEC is associated with intestinal hypoperfusion and hypoxia secondary to poor cardiac output, cyanosis, and pulmonary overcirculation. The most common congenital cardiac malformations that can lead to this disease are HLHS, systemic obstructive lesions, and defects with diastolic runoff. The incidence of this intestinal complication in cardiac patients ranges from 1.6% to 9% and is as high as 11% to 20% in patients with HLHS [2,67,124,125]. Low birth weight also increases the risk of this pathology [125,126] and more recent studies have shown that low diastolic blood pressure in ductal-dependent lesions may play a role [127]. Although most cases of cardiac NEC are mild, its presence may predict a poor prognosis for the patient’s future, prolong hospital length of stay, and even increase mortality [68,128,129].

Cardiac NEC classically presents with hematochezia and/or abdominal distension (Table 1). Hypotension, tachycardia, emesis, respiratory distress, apnea, and metabolic acidosis may also be present. Most cases occur during the postoperative stage [128,130] and predominantly affect the terminal ileum, cecum, and colon [131]. Currently, it is diagnosed through abdominal radiograph, which demonstrates intestinal pneumatosis, portal pneumatosis, intestinal dilatation, or, in more advanced stages, pneumoperitoneum [132]. Recently, abdominal ultrasound has been identified as a possible effective tool for diagnosing this entity [133]. Although controversy remains, recent studies have found no association between the initiation or augmentation of enteral nutrition and the development of cardiac NEC [23,49,54,56,63,64,69,130,134,135,136,137,138]. Some investigators have found that breast milk, human milk fortifiers, and the use of enteral nutrition protocols may decrease the risk of cardiac NEC in both experimental animal models and human patients [26,80,124,139,140,141]. Treatment includes a period of fasting and the administration of antibiotics (active against enteric pathogens), which can range from 48 h to 14 days [79]. However, it is essential to acknowledge that prolonged NPO status and antimicrobial therapy can alter the intestinal microbiome, potentially increasing the risk of developing cardiac NEC [79,142]. Although it rarely requires surgical intervention, consultation with a pediatric surgical team is often recommended.

### 8.2. Protein-Losing Enteropathy

Protein-losing enteropathy is a condition in which large volumes of protein leak through the intestinal mucosa and are lost in the feces, causing hypoproteinemia, chronic diarrhea, ascites, and edema [43,79,143]. This increase in intestinal permeability is secondary to intestinal congestion and an elevation in mesenteric vascular resistance, which results in damage to the mucosal epithelium and enlargement of the intestinal lymphatics [79,144]. This complication is more common in patients with failed Fontan physiology and occurs with a prevalence ranging from 3.7% to 24% [145]. The presence of alpha-1 antitrypsin in a stool sample confirms the diagnosis. Management is based on interventions that ameliorate hemodynamic disturbances and nutritional therapy that increases protein administration up to 3 g/kg/day. Occasionally, nasogastric tube feeding or parenteral nutrition may be required to achieve these nutritional goals [146]. Some clinicians also recommend using diets rich in medium-chain triglycerides, which are directly absorbed into the portal system, with the goal of decreasing lymphatic pressure and protein leakage [147].

## 9. Conclusions

Malnutrition increases morbidity and mortality in pediatric cardiac patients. Optimization of nutritional therapy plays a crucial role in the management of these infants and children. Randomized control trials that identify the most efficient perioperative feeding algorithms and cardiac NEC prevention and treatment strategies should be the focus of future research in the field. These studies would help standardize nutritional therapy and decrease variability. Until then, physicians, nurses, and dieticians should implement the most recent clinical nutritional guidelines to improve outcomes in CHD and heart failure.

## Figures and Tables

**Table 1:** Diagnosis and management of cardiac necrotizing enterocolitis ^1^.

Physical Exam	- Hematochezia - Abdominal distension - Hypotension - Tachycardia	- Emesis - Respiratory distress - Apnea - Irritability
Imaging	Chest X-ray: - Intestinal pneumatosis - Portal pneumatosis - Intestinal distension - Pneumoperitoneum	Abdominal ultrasound: - Intestinal wall thickening - Ascites - Portal pneumatosis - Pneumoperitoneum
Laboratory Studies	- Blood culture - Lactate (>2 mmol/L) - C reactive protein (>4 mg/dL) - White blood cell count (>20,000/μL)	
Treatment	Negative laboratory studies: - NPO for 48 h - Cefepime - Metronidazole	Positive laboratory studies: - NPO for 5–10 days - Cefepime - Metronidazole

^1^ Adapted from the protocols and clinical guidelines of the cardiac intensive care unit of Children’s Mercy Hospital, Kansas City, MO, USA.

## Supplementary Materials

The following supporting information can be downloaded at: https://www.mdpi.com/article/10.3390/nu17223609/s1, Figure S1: Flow chart demonstrating method of comprehensive literature search and reference selection.

## Abbreviations

The following abbreviations are used in this manuscript:

CHD	Congenital heart disease
NEC	Necrotizing enterocolitis
IC	Indirect calorimetry
REE	Resting energy expenditure
ECMO	Extracorporeal membrane oxygenation
HLHS	Hypoplastic left heart syndrome
BMI	Body mass index
VAD	Ventricular assist device
