# Supplementary figures and images for: Perioperative Nutrition in Pediatric Patients with Congenital Heart Disease and Heart Failure
Source: Nutrients. 2025 Nov 19;17(22):3609. doi: 10.3390/nu17223609 (PMC12655550; doi:10.3390/nu17223609)

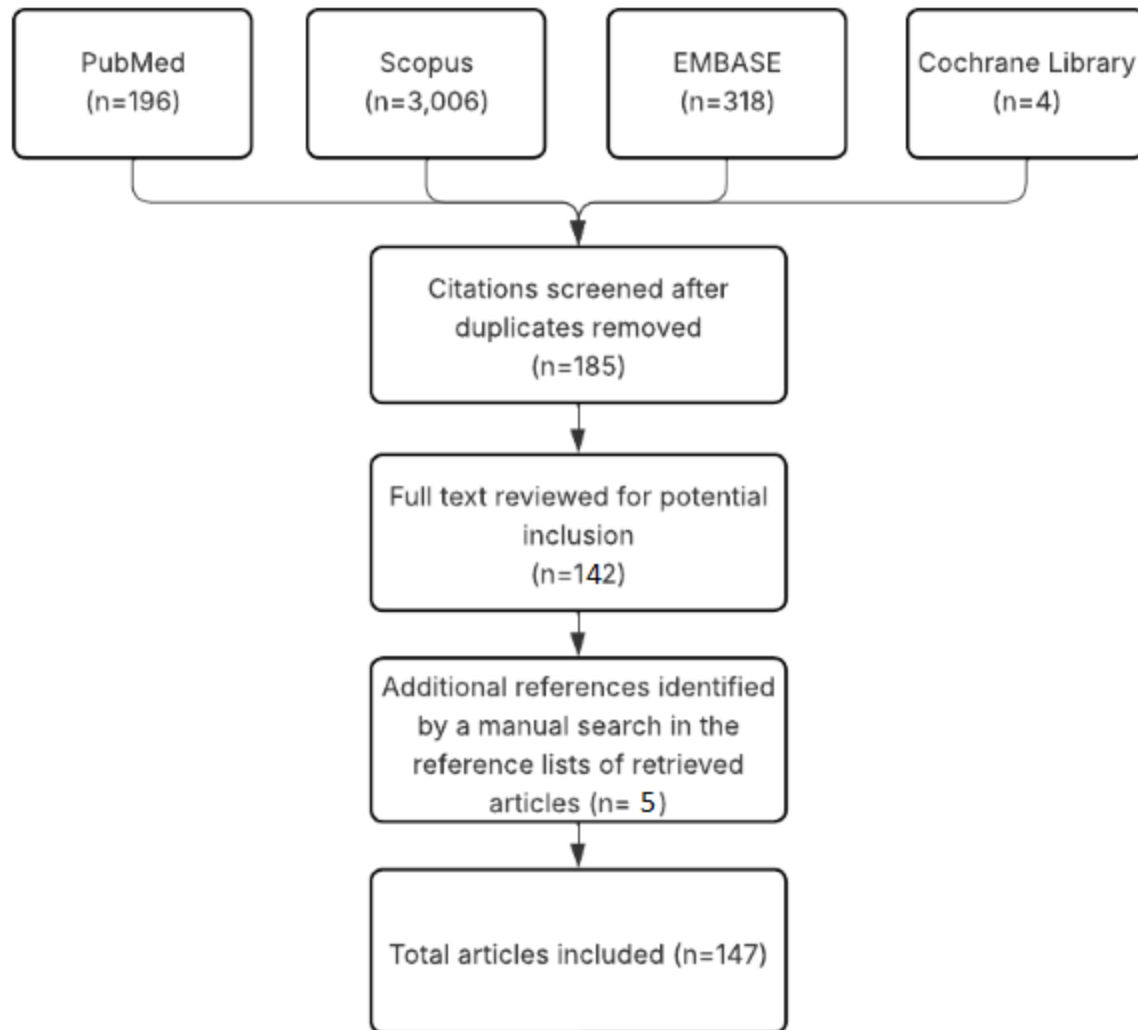

**Figure S1.** Flow chart of the literature search and reference selection process.

Supplement: Supplementary file 1 [file nutrients-17-03609-s001.zip › nutrients-3944100-supplementary.pdf]
